# Comparison of adsorptive capacity for different types of activated charcoal for common veterinary toxicants

**DOI:** 10.3389/fvets.2026.1741145

**Published:** 2026-02-18

**Authors:** Anika Chaganti, Benjamin M. Brainard

**Affiliations:** Department of Small Animal Medicine and Surgery, College of Veterinary Medicine, University of Georgia, Athens, GA, United States

**Keywords:** activated charcoal, adsorption, intoxication, veterinary, veterinary emergency and critical care

## Abstract

Activated charcoal (AC) is a convenient, effective method for gastrointestinal decontamination, typically supplied as either activated charcoal powder (pAC) or proprietary mixtures in granules or suspension (e.g., Toxiban^®^, Tox). We compared the adsorptive capacity for common veterinary toxicants of a new resin-bound activated charcoal product (Ready Rescue™; RR) to PAC and Tox. We hypothesized that RR is equivalent to Tox and PAC in binding of these toxicants. Solutions of simulated low pH (pH = 1.5) and neutral (pH 6–7) environments were incubated at 38 °C with continuous agitation. Commercial products containing the following toxicants (final concentration) were evaluated: Naproxen (8.8 mg/mL), ivermectin (1.1 mg/mL), bromethalin (0.028 mg/mL), xylitol (17.9 mg/mL), ethylene glycol (400 mg/mL), Delta-9 THC (1.5 mg/mL), baker’s chocolate (567 mg/mL), roquefortine (500 mg/mL of cheese), tartaric acid (133 mg/mL). Toxicants were incubated in separate reaction mixtures for 30 min before the equivalent of 15 g of charcoal product (RR, PAC, or Tox) was added. The reaction mixtures were sampled prior to charcoal addition, and at 30, 60, and 240 min after, and assayed for toxicant concentration. The different AC formulations decreased concentrations of the assayed toxicant in most tested conditions, in both acidic and neutral pH environments. Exceptions included tartaric acid, bromethalin, ethylene glycol, and xylitol, which showed variable changes in concentration. In general, the rate of decrease was similar between AC products with the exception of Delta-9 THC in the neutral environment, where Tox showed a more rapid rate of decrease than RR. In this *in vitro* system, the three tested AC products effectively decreased concentration of most toxicants over a 4 h period, with pAC and Tox showing a more rapid rate of decline for some toxicants compared to RR.

## Introduction

Activated charcoal (AC) administration is a convenient and effective method for gastrointestinal decontamination in veterinary medicine. Intoxication is a common veterinary emergency with 451,000 calls to the ASPCA poison control center made in 2024 alone (1). The top ten categories of small animal toxicants reported in 2024 included prescription and over-the-counter medications, human food and drinks, plants and fungi, rodenticides, insecticides, household products, and recreational drugs (1). Depending on the toxicant, the administration of AC early after ingestion can slow or prevent systemic absorption of the toxicant and limit clinical signs of intoxication (2).

Activated charcoal is produced by heating charcoal and then exposing it to steam to create an internal pore structure that promotes adsorption of various toxins through Van der Waals interactions (3). The internal pore structure results in a surface area between 900–2,000 m^2^/g that facilitates adsorption of various compounds (3). Activated charcoal has a higher affinity for larger, lipophilic molecules with a neutral charge, and does not readily adsorb heavy metals, electrolytes, alcohols, strong acids, or strong bases (4). Activated charcoal is supplied as either granules or a powder, or is available as proprietary suspensions. In addition to AC, these proprietary mixtures may contain other compounds such as kaolin and sorbitol (5). Dosing recommendations for activated charcoal products are administration at a 10:1 weight ratio (for mg of ingested toxin) in cases where the dose of toxin is known (4), or they can be empirically dosed at 1–2 g/kg body weight PO (5).

A new commercially available AC product has been created using a resin-based process that results in the formation of spherical granules of AC. These granules allow the product to be more compact but still retain a surface area of 1,362 m^2^/g because of the porous nature of the AC (Personal communication with Matthieu Glassman VMD, DACVS, Allpet Inc/Dr. Cuddles). Compared to other AC products, the spherical form has an increased density resulting in a smaller volume (approximately 1/3 of non-spherical products) for the same weight of AC. Initial *in vitro* testing with acetaminophen supports the adsorptive capacity of the new product (Personal communication with Matthieu Glassman VMD, DACVS, Allpet Inc/Dr. Cuddles), however, the resin sphere AC has not been tested for adsorption of other common canine toxicants.

The objective of this survey study was to compare the adsorptive capacity of the resin-based AC product (Ready Rescue™; RR) to two other AC products (ToxiBan^®^ [Tox]) and chemical grade activated charcoal (Fisher Scientific, [pAC]) for several common small animal toxicants. We hypothesized that RR would have equivalent binding of the selected toxins as Tox and pAC.

## Materials and methods

This pilot study was designed to perform a broad survey of the adsorptive capacity of different types of AC for common veterinary toxicants. Simulated gastric and intestinal environments were prepared using commercially available media (FaSSGF/FaSSiF, Biorelevant) according to the manufacturer instructions (6). Briefly, the supplied mixture of powdered enzymes and bile salts was added to a diluted buffer concentrate and mixed thoroughly. The solutions were pH tested before experimental use, with the simulated stomach environment (“acidic pH”) having a pH between 1–2 and the simulated intestinal environment (“neutral pH”) a pH between 6–7. Although the fasted canine stomach has a slightly alkaline environment, the actively digesting stomach is acidic, and the toxicant products were rationalized to be ingested under both scenarios (7).

### Charcoal preparation

Charcoal products were measured to provide an equal weight of AC across products. This resulted in a dose of 15 g of RR, 30 g of Tox granules (15 g of AC, accounting for approximately 15 g of kaolin and sorbitol), and 15 grams of pAC. For some of the latter experiments, a product backorder made it difficult to obtain the granular form of Tox and so the commercial liquid form was substituted, resulting in the addition of 150 mL of ToxiBan^®^ suspension (15 g equivalent of charcoal, without sorbitol). The dose of 15 g was chosen based on a hypothetical initial dosing of a 10 kg dog (reflecting an approximate 1.5 g/kg of charcoal) as well as practical consideration after a larger dose of tox granules (60 g total) solidified the 100 mL reaction mixture. All toxicants were tested with Tox granules except for Δ-9 THC, Baker’s chocolate, tartaric acid, and roquefortine. The final assayed concentration of compounds in the flasks receiving the 150 mL of Tox suspension were adjusted for the increased volume of the flasks following addition (i.e., the baseline concentrations were not changed, but the subsequent concentrations as reported were scaled to account for dilution in 250 mL vs. 100 mL).

### Experimental protocol

For each assay, 1 L Erlenmeyer flasks were filled with 100 mL of either the acidic solution or the neutral pH solution and held in a 38 °C water bath for at least 10 min prior to toxicant addition. The platform holding the flasks was continuously agitated with an orbital mixer. Following toxicant addition, flasks were maintained under these conditions for 30 min to allow for dissolution and dissociation of toxicants. After this incubation period, a 10 mL aliquot of reaction mixture was collected (baseline). Subsequently, one of the three charcoal products was added to individual flasks and incubation continued as above. Subsequent samples were collected 30, 60, and 240 min after addition of the AC.

The 10 mL aliquots of reaction mixture were centrifuged for 5 min at 978×*g*, followed by an additional centrifugation of the supernatant at 16,000×*g* for 2 min. The supernatant was then stored at −80 °C for no longer than 2 months prior to analysis by specific reference laboratories.

### Toxicants

Toxicants studied included Naproxen (Aleve tablets, Bayer Healthcare, Whippany, NJ) at a concentration of 8.8 mg/mL, ivermectin (Durvet paste 1.87%, Bimeda-MTC Animal Health Inc., Cambridge, Ontario, Canada) at a concentration of 1.1 mg/mL, and bromethalin (Motomco Tomcat Mouse Killer blocks, 0.01%, Madison WI) at a concentration of 0.028 mg/mL. Sugar free xylitol-containing gum (Sugar Free Trident Spearmint Gum sticks, Mondelez Global LLC, East Hanover NJ) was added at a concentration of 17.9 mg/mL (8). Ethylene glycol (Sigma-Aldrich, Belgium) was added to a concentration of 400 mg/mL. Delta-9 THC (Palmetto Hemp Supply S’MORES Bars brownie bar, 300 mg Delta-9, Charleston SC) was added to a predicted concentration of 1.5 mg/mL, assuming equal distribution of the Delta-9 THC throughout the bar, which was weighed and then divided equally by weight between flasks. Chocolate (Baker’s Premium Baking Bar Unsweetened Chocolate 100% Cacao, Chicago IL) was added to 567 mg/mL. To study adsorption of roquefortine, Roquefort cheese (Murray’s cheese, New York, NY) was incubated at an ambient temperature of approximately 24 °C for a week, prior to addition at a concentration of 500 mg of cheese/mL (again divided by weight). Tartaric acid (Great value, Bentonville, AR) was added to a concentration of 133 mg/mL. No attempt was made to break up any toxicant or to mimic mastication.

### Analytical methods

Sample analysis was carried out at various reference laboratories using different methodologies. Naproxen concentration and ivermectin concentrations were analyzed using HPLC (Clinical Pharmacology Laboratory, North Carolina State University, Raleigh NC). Bromethalin, xylitol, and tartaric acid concentrations were assayed using liquid chromatography-multiple reaction monitoring-mass spectrometry (LC-MRM/MS, Creative Proteomics, Shirley, NY). Delta-9 THC concentrations were quantified by gas chromatography tandem mass spectrometry (GC–MS/MS) using multiple reaction monitoring against a certified laboratory standard (Michigan State University Veterinary Diagnostic Laboratory). Ethylene glycol concentrations were assayed using gas chromatography–mass spectrometry (GC–MS), and methylxanthines (theobromine, caffeine) and roquefortine were assayed using LC–MS (Pennsylvania Animal Diagnostic Laboratory, Kennett Square, PA). With the exception of roquefortine, all assayed concentrations were within the quantification limits for the individual assays, or reported as 0 if no sample was detected (e.g., ivermectin).

### Statistical methods

Raw concentration data for each timepoint and between each AC product were compared using a 2-way ANOVA (Prism, GraphPad Software v. 10). When differences were identified between AC types, a Tukey’s test for multiple comparisons was performed to compare individual types. Because the measured starting concentrations were different between each flask, data is displayed as a percentage of the starting concentration, which was characterized as 100%. Statistical significance was set at a *p* value < 0.05. The measured concentrations are provided as Supplementary material.

## Results

Concentrations for each toxicant except roqufortine are tabulated by flask pH (acid/neutral) and by AC product. To facilitate comparison between AC products, the concentrations were normalized to 100% as a starting concentration, because the degree of dissolution at the baseline measurement was variable between flasks, although statistical analysis was performed on the raw concentration data, as indicated above. The values for the RR group are summarized in Tables 1, 2, for pAC in Tables 3, 4, and for TOX in Tables 5, 6. The measured concentrations of toxicants are appended using the same organization as Supplementary material.

**Table 1:** Percentage change in concentration of assayed toxicants after exposure to ReadyRescue (RR) activated charcoal product in an acidic environment.

Time	Toxicant								
	Naproxen	Ivermectin	Bromethalin	Ethylene glycol	Xylitol	Tartaric acid	Theobromine	Caffeine	Delta-9 THC
0 min	100	100	100	100	100	100	100	100	100
30 min	1.66579906	1.93823916	39.37008	85.8895706	139.839265	138.036765	46.1538462	53.33333	14.9789
60 min	0.3539823	0.19710907	30.67485	46.0736196	211.050517	181.226468	38.0769231	42.66667	16.87764
240 min	0.10411244	0.39421813	57.47126	54.9693252	354.764638	246.044245	28.8461538	26.66667	75.61181

The baseline concentrations (0 min) are normalized to represent 100%, with subsequent timepoints being the remaining percentage of toxicant in the supernatant of the flask.

**Table 2:** Percentage change in concentration of assayed toxicants after exposure to ReadyRescue (RR) activated charcoal product in a neutral pH environment.

Time	Toxicant								
	Naproxen	Ivermectin	Bromethalin	Ethylene glycol	Xylitol	Tartaric acid	Theobromine	Caffeine	Delta-9 THC
0 min	100	100	100	100	100	100	100	100	100
30 min	3.61224778	37.470701	212.1086	109.146341	172.216117	369.002504	53.84615	66.42857	83.02034
60 min	0.97361366	16.322182	146.3977	25.9146341	162.600733	404.598362	46.15385	55	92.8795
240 min	0.05644137	0.29831664	240.1891	50.3658537	303.846154	459.602761	29.23077	27.14286	64.63224

The baseline concentrations (0 min) are normalized to represent 100%, with subsequent timepoints being the remaining percentage of toxicant in the supernatant of the flask.

**Table 3:** Percentage change in concentration of assayed toxicants after exposure to pure activated charcoal (pAC) in an acidic environment.

Time	Toxicant								
	Naproxen	Ivermectin	Bromethalin	Ethylene glycol	Xylitol	Tartaric acid	Theobromine	Caffeine	Delta-9 THC
0 min	100	100	100	100	n/a	100	100	100	100
30 min	0.0243135	0	78.125	128.506787	n/a	245.038746	5	8.125	7.375328
60 min	0.01716247	0	100	262.443439	n/a	146.83306	4.230769	7.5	4.593176
240 min	0.01258581	0	35.21127	19.306184	n/a	143.518224	3.846154	6.875	3.43832

The baseline concentrations (0 min) are normalized to represent 100%, with subsequent timepoints being the remaining percentage of toxicant in the supernatant of the flask.

**Table 4:** Percentage change in concentration of assayed toxicants after exposure to pure activated charcoal (pAC) in a neutral pH environment.

Time	Toxicant								
	Naproxen	Ivermectin	Bromethalin	Ethylene glycol	Xylitol	Tartaric acid	Theobromine	Caffeine	Delta-9 THC
0 min	100	100	100	100	100	100	100	100	100
30 min	0.00197857	0	21.22642	62.9834254	100.451637	256.550346	5.357143	7.142857	14.44043
60 min	0.00197857	0	67.92453	91.7127072	100.200728	320.098386	4.285714	5.714286	16.17329
240 min	0.00544106	0	163.6364	127.624309	75.6994104	298.625673	3.928571	5.238095	23.2491

The baseline concentrations (0 min) are normalized to represent 100%, with subsequent timepoints being the remaining percentage of toxicant in the supernatant of the flask.

**Table 5:** Percentage change in concentration of assayed toxicants after exposure to Toxiban (Tox) activated charcoal product in an acidic environment.

Time	Toxicant								
	Naproxen	Ivermectin	Bromethalin	Ethylene glycol	Xylitol	Tartaric acid	Theobromine	Caffeine	Delta-9 THC
0 min	100	100	100	100	100	100	100	100	100
30 min	0.0726477	0	58.82353	256.227758	247.804878	37.7980735	5.454545	7.857143	0.770432
60 min	0.01400438	0	118.3432	143.950178	484.634146	34.688124	11.81818	10	0.754815
240 min	0.00962801	0	72.20217	161.565836	1235.36585	35.900975	16.36364	12.14286	5.98126

The baseline concentrations (0 min) are normalized to represent 100%, with subsequent timepoints being the remaining percentage of toxicant in the supernatant of the flask.

**Table 6:** Percentage change in concentration of assayed toxicants after exposure to Toxiban (Tox) activated charcoal product in a neutral pH environment.

Time	Toxicant								
	Naproxen	Ivermectin	Bromethalin	Ethylene glycol	Xylitol	Tartaric acid	Theobromine	Caffeine	Delta-9 THC
0 min	100	100	100	100	100	100	100	100	100
30 min	0.05039012	0.73184462	52.65957	132.234957	297.167585	61.2898164	9.52381	10	0.123894
60 min	0.01625488	0.15012197	5.003791	137.106017	453.422502	103.696846	16.19048	13.07692	0.029499
240 min	0.03738622	0.11259148	17.06897	142.836676	662.470496	62.5105916	17.14286	13.84615	3

The baseline concentrations (0 min) are normalized to represent 100%, with subsequent timepoints being the remaining percentage of toxicant in the supernatant of the flask.

Naproxen was adsorbed by all three AC products, and all concentrations decreased with time (*p* < 0.001). There was no difference in the degree of decrease for AC product in either the acidic solution (*p* = 0.2349) or neutral pH solution (*p* = 0.2369). Ivermectin also showed a significant decrease with time (*p* < 0.001) but there was also no difference in the rate of decrease of ivermectin in the acidic or neutral pH fluid between the 3 different types of AC (*p* = 0.2108, 0.1778, respectively).

Bromethalin in the acidic solution was not well adsorbed by any AC product. Over the 4 h of the study, there was no difference from baseline in the bromethalin concentration in any group (*p* = 0.5349). Bromethalin concentrations in the neutral pH solution were most prominently decreased in the Tox group, with a 5% residual concentration after 60 min. In the neutral pH solution, the RR group displayed increasing concentrations, while the AC group showed an initial drop which rose to a concentration above baseline by 4 h. Neither the rate of decrease nor the type of AC was significantly different from the others, however (*p* = 0.6432, *p* = 0.1977, respectively).

Theobromine in acidic solution was adsorbed by all AC products (*p* = 0.001). There was not a significant difference in the rate of concentration decrease between AC types (*p* = 0.086). Similarly, in the neutral pH fluid, there was a significant change in theobromine over time (*p* = 0.004), and there was no difference in the rate of decrease between AC types (*p* = 0.1454). Caffeine from the chocolate in the acid pH was also adsorbed by all activated charcoal products over time (*p* = 0.007). There was a not a significant difference in the rate of decrease of caffeine between AC types (*p* = 0.1068). In the neutral pH the concentration of caffeine decreased over time (*p* = 0.016) and no difference between AC types was identified (*p* = 0.5824).

Roquefortine appeared to be adsorbed by all three AC products, although relatively little overall compound was eluted from the cheese. All concentrations were below the listed reporting limit of 0.01 μg/mL, however extrapolation of the detected signal intensities to estimate concentration demonstrated a detectable baseline roquefortine that became essentially undetectable in subsequent assays (see Supplementary material).

Delta-9 THC was adsorbed by all AC products (*p* = 0.0025). In acidic solution, there was no difference between rate of decrease with AC type (*p* = 0.1860). In the neutral pH fluid, there was not a significant decrease in concentration with time (*p* = 0.0547) and no difference in adsorption between AC types (*p* = 0.9448).

Ethylene glycol was not adsorbed by any AC products in acidic pH (*p* = 0.5295), with actual increases seen in concentration in the AC and TOX solution over 4 h, but with no difference between charcoal types (*p* = 0.2690). In the neutral pH, there was also no decrease in concentration with time (*p* = 0.8772), and no difference in rate between AC types (*p* = 0.1455).

Due to an error in the baseline measurement of xylitol in acid pH treated with pAC, the serial comparison was only performed for the RR vs. the Tox group. Xylitol was not adsorbed by either RR or Tox, and the concentration did not change over time in the acidic solution (*p* = 0.2494). There was also no difference in adsorptive capacity between RR and Tox (*p* = 0.0653). In the neutral pH solution, there was no change in xylitol concentration over time (*p* = 0.2515) despite apparent increases in both the RR and Tox groups over 4 h. There was no difference seen in the rate of concentration change between AC types (*p* = 0.1088).

Tartaric acid was also not adsorbed by the tested AC products, with the concentration of tartaric acid remaining stable over time (*p* = 0.4934). There were no differences in the change in concentration of tartaric acid for each AC type (*p* = 0.0917). Over the 4 h of the study, there was an increase in the concentration of tartaric acid in the neutral pH solution (*p* = 0.0136), and there was a significant difference between the rates of change of tartaric acid concentration between RR and Tox (*p* = 0.0330).

## Discussion

The different AC formulations tested herein similarly decreased concentrations of the majority of assayed toxicant in simulated gastrointestinal fluid with both acidic and neutral pH. Within 30 min of addition of all AC products, more than 96% of naproxen and 98% of ivermectin was adsorbed in both environments. Naproxen ingestion above 25 mg/kg is expected to result in renal toxicity in dogs, and we used a total of 880 mg in the flask (9). The tested dose of ivermectin was 114 mg, and severe neurologic symptoms are expected to manifest at doses between 1–2.5 mg/kg in dogs with a functional MDR gene (and result in more severe toxicity in a dog with non-functional MDR gene) (10). While the tested dosage of 15 g of AC is consistent with a dose that might be administered to a 10 kg. dog, almost all toxicant was adsorbed in the test flasks. While RR and pAC contain only AC, Tox is a proprietary mixture of kaolin and AC, supplied either as a liquid with 10.4% AC and 6.25% kaolin, or as granules containing 47.5% AC, 10% kaolin, and 20% sorbitol. Based on the results shown here, we suspect that administration of AC products after oral consumption of naproxen or ivermectin would adsorb the majority of remaining drug in the stomach and intestine.

Delta-9 THC was also adsorbed to an equivalent degree by all AC types in both acidic and neutral pH environments. The LD_50_ for THC in dogs has not been determined, and the amount of active ingredient in many products can be variable and unpredictable (11). We chose a baked product with a stated amount of Δ-9 THC, but the chances that the compound was evenly distributed throughout the brownie are not known. This is important because equivalent weight pieces were divided in thirds for the assays, but variation in Δ-9 THC content for each specific piece was not known.

Bromethalin was less well adsorbed over the study time period compared to other toxicants, but all AC products adsorbed the bromethalin equivalently in both acidic and neutral pH environments. The persistent bromethalin detected in the treated flasks may have been a result of continued elution from the bait blocks that were used as a source, as they were not removed prior to introduction of the AC. We used 2.8 mg of bromethalin in each assay which was the amount contained in a single block of rodenticide [the LD_50_ of bromethalin is 2.38–5.6 mg/kg in dogs (12)], and the blocks were not crushed prior to addition (although they visually appeared to dissociate), which may have contributed to the prolonged elution of bromethalin. An additional possibility for the lack of significant decrease in bromethalin concentration can also be due to binding and subsequent dissociation of the toxicant from the AC; we did not study the kinetics of binding of any toxicant to AC but this may also have contributed to the spikes in bromethalin concentration seen at the 60 and 90 min timepoints for some AC types.

Differences in the rate of adsorption between AC types were seen for theobromine and caffeine derived from baker’s chocolate. The flasks with RR demonstrated a slower rate of decrease in concentration compared to that of Tox or pAC, specifically slower than pAC for theobromine in the acidic environment and Tox for theobromine in the neutral pH. Caffeine in the acid pH was adsorbed similarly by all activated charcoal products after 4 h, but the rate of caffeine concentration decrease was more rapid in pAC compared to RR. In the neutral pH the concentration of caffeine decreased in a similar manner, but any differences in rate between the AC products did not reach statistical significance. Theobromine and caffeine as tested here were components of baker’s chocolate, in this case administered as 56.7 g of chocolate (13). Given that chocolate also contains cocoa butter and other large molecules, the real and apparent slower adsorption in the RR group may also represent steric hindrance or coating of the granules by the fats in the compound. It will be beneficial to make further observations of adsorption efficacy in the presence of possible interfering substances, and it is probably wise to avoid administering RR to dogs using fatty admixtures (e.g., peanut butter) as opposed to mixing with water or possibly small amounts of food, to avoid any possible obstacle for access to the interior pore structure. The molecular weights of caffeine and theobromine are smaller than other compounds that were adsorbed well by RR (e.g., ivermectin, see Table 7), so we do not believe that the size of the molecules played a role in the slower adsorption.

**Table 7:** Molecular weights, octanol/water partition coefficient (logP), and dissociation constants (pK), of assayed toxicants.

	Toxicant									
	Naproxen	Ivermectin	Bromethalin	Roquefortine	Ethylene glycol	Xylitol	Tartaric acid	Theobromine	Caffeine	Delta-9 THC
Molecular weight	230.26 g/mol (17)	875.1 g/mol (18)	577.93 g/mol (19)	389.4 g/mol (20)	67.07 g/mol (21)	152.15 g/mol (22)	150.09 g/mol (23)	180.16 g/mol (24)	194.19 g/mol (25)	314.5 g/mol (26)
logP	3.3 (17)	4.1 (18)	6.2 (19)	3 (27)	−1.4 (21)	−2.5 (22)	−1.9 (28)	−0.8 (24)	−0.1 (25)	7 (26)
pKa	4.15 (17)	12.47 (29)	9.0 (19)	11.10 (20)	15.1 (21)	13.24 (22)	pKa1 = 2.98 pKa2 = 4.34 (23)	9.9 (24)	14 (25)	10.6 (26)

Due to low concentrations, the adsorption of roquefortine was not able to be compared between AC types, and the only data is based on extrapolation of signal that was below the reporting limit of the laboratory that ran the assay. While there appeared to be decrease in concentration in all assays of roquefortine (from detectable to undetectable), caution is warranted in extrapolating this information to clinical cases. The toxic dose of tremorgenic mycotoxins such as roquefortine in dogs has not been identified (14), and we used 50 mg of aged Roquefort cheese in each trial. While the source was authentic Roquefort cheese, additional trials with larger doses of pure toxicant are indicated, both as an improved assay and to verify equal amounts of toxicant to each assay (assuming that the roquefortine was not evenly distributed throughout the cheese).

In this study, the source toxicant compounds were chosen to mimic an actual animal ingestion, instead of studying adsorption of isolated toxicants. Although this was a more realistic usage of the products, the impact of excipient products (e.g., the cocoa butter in chocolate) that may have altered the adsorption kinetics could not be accounted for. Similarly, we suspect that the continued increase in concentration of toxicant in some assays represented continued elution of toxicant from the source compound, rather than a representation of saturation of charcoal binding sites on the AC products, but this must be considered as another possibility. Likewise, depending on the compounding and the specific assay, we cannot rule out interference from the components of the toxicants on the assays. Specifically, some components of the ivermectin paste were detected along with the ivermectin on the HPLC output from the assays, and a more specific assay (e.g., GC–MS) may be less likely to demonstrate interference. Future testing to determine actual adsorptive efficacy should be focused on comparisons between known quantities of pure compounds.

All of the tested compounds had molecular weights (MW) between 67.07 g/mol and 875.1 g/mol. Though there is no published report on the exact size range of molecules activated charcoal binds, previous studies have utilized compounds with molecular weights between 100–800 g/mol (15). The binding of compounds to AC is thought to be through intermolecular or van der Walls forces. Van der Walls forces describe attraction of compounds without any change in the structure of the compound and can occur between both uncharged and charged molecules, with charged molecules potentially able to form stronger bonds with the uncharged AC. The impact of assay pH on the ionization of the compounds may thus have impacted the tightness of binding to the AC molecules and ability to saturate available binding sites, which could have impacted the apparent adsorption as measured here. Additionally, the overall solubility of some compounds in aqueous solution may have hindered dissociation of individual compounds, although the acid and neutral fluids did contain bile salts and other emulsifiers as might be found in stomach or intestinal secretions, and all toxicants were able to be detected on the assays, with only roquefortine being found at very low concentrations. The relative lipophilicity (as reflected in the octanol–water partition coefficient, logP) of the tested compounds is also listed in Table 7. Although the lipophilic compounds (logP>3) did exhibit binding to all AC formulations, as expected, some non-lipophilic compounds (e.g., caffeine, theobromine) also showed significant adsorption, which may be a factor of other molecular characteristics (e.g., molecular geometry).

The assayed alcohols, xylitol and ethylene glycol, were not expected to show evidence of adsorption to charcoal. The increasing xylitol concentration at the 4 h points was hypothesized to be due to continued elution from the gum, as it dissolved more slowly than other toxicants. Although it was expected that the ethylene glycol concentrations would not decrease, the RR and AC products did show decreased concentrations after longer incubation in both the acidic and neutral pH environments. Similarly, the tartaric acid concentrations, which increased in both pH environments exposed to RR and AC, showed a decrease to about 30% of baseline in the Tox group with acidic pH, and to 60% of baseline in the neutral pH environment. These results, as well as the decrease in the assays with some of the alcohols, may be due to the presence of kaolin in the Tox mixture, or due to the ionization of tartaric acid in the neutral pH. Tartaric acid was chosen for study due to its putative implication in the renal toxicity caused by ingestion of grapes, raisins, or tamarind (16). Kaolin is a clay composed of aluminum silicates that is useful for binding of compounds in the gastrointestinal tract but does not create chemical bonds in the same manner as AC, rather resulting in bulk sequestration of toxins and other compounds. Because of the relative lack of specificity of kaolin, it may have an enhanced ability to bind alcohols, and may work synergistically with AC.

Ready Rescue has a similar surface area as Tox or AC but a higher density, with part of the surface area contained within the spherical granules. The exact surface area of the Tox and pAC that were tested in this investigation is not known, and so the actual adsorptive surface area per weight of AC may have been different. We also encountered a backorder of Tox, which forced us to change products from the granules to the solution midway through this experiment. Notably, the granules contain 10% Kaolin, 20% Sorbitol and 22.5% unidentified wetting and dispersing agents in addition to 47.5% AC, while the tested suspension contained only 10.4% AC and 6.25% kaolin in an aqueous base. While the application was based only on equivalent amounts of AC, we cannot predict the impact of the other ingredients on adsorption of toxins between the two Tox products.

Other limitations include the fact that due to logistics and funding, only single adsorption trials were performed for each toxicant and timepoint. Optimally these would have been performed in replicates. Based on the results seen here, future studies can focus primarily on the 30 and 60 min timepoints, especially because the toxicant will likely be absorbed or moved out of the stomach at the 4 h time point under circumstances of an *in vivo* unplanned ingestion. Additionally, to mimic an actual pet ingestion scenario, commercially available products, in various substrates or vehicles were tested. To obtain specific information about the exact ability of the different AC products to adsorb the tested toxicants, pure compound should be used. Using a set amount of pure compound will also simplify data analysis, as it is suspected that the continued increase in concentration of some toxicants was related to continued elution of the compound from the substrate. Due to the lack of pure toxicant used here, we also cannot predict the impact of the specific preparations on the analysis. Although samples were grossly purified through centrifugation prior to analysis, interfering compounds from the toxicant vehicles may have caused some degree of interference in the final assays.

In addition, the impact of admixture of any AC with food (as is commonly used to encourage ingestion of AC in pets) was not evaluated. Depending on the composition of the dog food, specifically of lipid soluble ingredients, it is plausible that the addition of lipid soluble components might impact adsorption of the actual toxin. One product (RR) has guidance that admixture with dog food does not impact adsorption while admixture using peanut butter may decrease toxicant adsorption. Whether this is true across AC types or if it is a function of the spherical shape of RR is not clear, and requires further study. Without further study, the investigators can only recommend administering an AC product admixed with water, or without admixture.

The *in vitro* data presented here suggests that RR has similar adsorptive capacity to Tox and pAC for common canine toxicants, but these in vitro results may not reflect *in vivo* effects, and further *in-vivo* testing would be necessary to confirm clinical adsorptive efficacy.

## Data Availability

The original contributions presented in the study are publicly available. This data can be found here: https://openscholar.uga.edu/record/27694?&ln=en.
