# Supplementary material for: Comparison of adsorptive capacity for different types of activated charcoal for common veterinary toxicants
Source: Front Vet Sci. 2026 Feb 18;13:1741145. doi: 10.3389/fvets.2026.1741145 (PMC12958355; doi:10.3389/fvets.2026.1741145)
Supplement: Supplementary file 1 [file Table_1.docx]

**Supplemental tables:**

Table S1a: Change in concentration of assayed toxicants after exposure to ReadyRescue (RR) activated charcoal product in an acidic environment. Values for roquefortine are extrapolated from observed signal intensities but are technically below the reporting limit of the assay (0.01 µg/mL).

| Time | Toxicant | | | | | | | | | |
| --- | --- | --- | --- | --- | --- | --- | --- | --- | --- | --- |
|  | Naproxen (µg/mL) | Ivermectin (µg/mL) | Bromethalin (µg/mL) | Roquefortine (µg/mL) | Ethylene Glycol (µg/mL) | Xylitol (µg/mL) | Tartaric Acid (µg/mL) | Theobromine (µg/mL) | Caffeine (µg/mL) | Delta-9 THC (ng/mL) |
| 0 min | 1921 | 60.88 | 0.05 | 0.006 | 163000 | 3.484 | 4.0637 | 2600 | 150 | 2370 |
| 30 min | 32 | 1.18 | 0.127 | <0.005 | 140000 | 4.872 | 5.6094 | 1200 | 80 | 355 |
| 60 min | 6.8 | 0.12 | 0.163 | <0.005 | 75100 | 7.353 | 7.3645 | 990 | 64 | 400 |
| 240 min | 2 | 0.24 | 0.087 | <0.005 | 89600 | 12.36 | 9.9985 | 750 | 40 | 1792 |

Table S1b: Change in concentration of assayed toxicants after exposure to ReadyRescue (RR) activated charcoal product in a neutral pH environment.

| Time | Toxicant | | | | | | | | | |
| --- | --- | --- | --- | --- | --- | --- | --- | --- | --- | --- |
|  | Naproxen (µg/mL) | Ivermectin (µg/mL) | Bromethalin (µg/mL) | Roquefortine (µg/mL) | Ethylene Glycol (µg/mL) | Xylitol (µg/mL) | Tartaric Acid (µg/mL) | Theobromine (µg/mL) | Caffeine (µg/mL) | Delta-9 THC (ng/mL) |
| 0 min | 7087 | 93.86 | 1.016 | 0.01 | 164000 | 5.46 | 2.9554 | 2600 | 140 | 12780 |
| 30 min | 256 | 35.17 | 0.479 | <0.005 | 179000 | 9.403 | 10.9055 | 1400 | 93 | 10610 |
| 60 min | 69 | 15.32 | 0.694 | <0.005 | 42500 | 8.878 | 11.9575 | 1200 | 77 | 11870 |
| 240 min | 4 | 0.28 | 0.423 | <0.005 | 82600 | 16.59 | 13.5831 | 760 | 38 | 8260 |

Table S2a: Change in concentration of assayed toxicants after exposure to pure activated charcoal (pAC) in an acidic environment.

| Time | Toxicant | | | | | | | | | |
| --- | --- | --- | --- | --- | --- | --- | --- | --- | --- | --- |
|  | Naproxen (µg/mL) | Ivermectin (µg/mL) | Bromethalin (µg/mL) | Roquefortine (µg/mL) | Ethylene Glycol (µg/mL) | Xylitol (µg/mL) | Tartaric Acid (µg/mL) | Theobromine (µg/mL) | Caffeine (µg/mL) | Delta-9 THC (ng/mL) |
| 0 min | 3496 | 67.43 | 0.05 | 0.008 | 66300 |  | 4.0907 | 2600 | 160 | 3810 |
| 30 min | 0.85 | 0 | 0.064 | <0.005 | 85200 | 10.32 | 10.0238 | 130 | 13 | 281 |
| 60 min | 0.6 | 0 | 0.05 | <0.005 | 174000 | 12.49 | 6.0065 | 110 | 12 | 175 |
| 240 min | 0.44 | 0 | 0.142 | <0.005 | 12800 | 9.393 | 5.8709 | 100 | 11 | 131 |

Table S2b: Change in concentration of assayed toxicants after exposure to pure activated charcoal (pAC) in a neutral pH environment.

| Time | Toxicant | | | | | | | | | |
| --- | --- | --- | --- | --- | --- | --- | --- | --- | --- | --- |
|  | Naproxen (µg/mL) | Ivermectin (µg/mL) | Bromethalin (µg/mL) | Roquefortine (µg/mL) | Ethylene Glycol (µg/mL) | Xylitol (µg/mL) | Tartaric Acid (µg/mL) | Theobromine (µg/mL) | Caffeine (µg/mL) | Delta-9 THC (ng/mL) |
| 0 min | 6065 | 50.04 | 0.18 | 0.005 | 181000 | 7.971 | 3.2525 | 2800 | 210 | 27700 |
| 30 min | 0.12 | 0 | 0.848 | <0.005 | 114000 | 8.007 | 8.3443 | 150 | 15 | 4000 |
| 60 min | 0.12 | 0 | 0.265 | <0.005 | 166000 | 7.987 | 10.4112 | 120 | 12 | 4480 |
| 240 min | 0.33 | 0 | 0.11 | <0.005 | 231000 | 6.034 | 9.7128 | 110 | 11 | 6440 |

Table S3a: Change in concentration of assayed toxicants after exposure to Toxiban (Tox) activated charcoal product in an acidic pH environment.

| Time | Toxicant | | | | | | | | | |
| --- | --- | --- | --- | --- | --- | --- | --- | --- | --- | --- |
|  | Naproxen (µg/mL) | Ivermectin (µg/mL) | Bromethalin (µg/mL) | Roquefortine (µg/mL) | Ethylene Glycol (µg/mL) | Xylitol (µg/mL) | Tartaric Acid (µg/mL) | Theobromine (µg/mL) | Caffeine (µg/mL) | Delta-9 THC (ng/mL) |
| 0 min | 2285 | 69.71 | 0.2 | 0.021 | 56200 | 0.41 | 3.4052 | 2200 | 140 | 19210 |
| 30 min | 1.66 | 0 | 0.34 | <0.005 | 144000 | 1.016 | 1.2871 | 120 | 11 | 148 |
| 60 min | 0.32 | 0 | 0.169 | <0.005 | 80900 | 1.987 | 1.1812 | 260 | 14 | 145 |
| 240 min | 0.22 | 0 | 0.277 | <0.005 | 90800 | 5.065 | 1.2225 | 360 | 17 | 1149 |

Table S3b: Change in concentration of assayed toxicants after exposure to Toxiban (Tox) activated charcoal product in a neutral pH environment.

| Time | Toxicant | | | | | | | | | |
| --- | --- | --- | --- | --- | --- | --- | --- | --- | --- | --- |
|  | Naproxen (µg/mL) | Ivermectin (µg/mL) | Bromethalin (µg/mL) | Roquefortine (µg/mL) | Ethylene Glycol (µg/mL) | Xylitol (µg/mL) | Tartaric Acid (µg/mL) | Theobromine (µg/mL) | Caffeine (µg/mL) | Delta-9 THC (ng/mL) |
| 0 min | 6152 | 53.29 | 0.099 | 0.005 | 69800 | 1.271 | 3.1865 | 2100 | 130 | 33900 |
| 30 min | 3.1 | 0.39 | 0.188 | <0.005 | 92300 | 3.777 | 1.953 | 200 | 13 | 42 |
| 60 min | 1 | 0.08 | 1.9785 | <0.005 | 95700 | 5.763 | 3.3043 | 340 | 17 | 10 |
| 240 min | 2.3 | 0.06 | 0.58 | 0 | 99700 | 8.42 | 1.9919 | 360 | 18 | 1017 |
